# The Federation of Medical and Allied Societies

**Published:** 1921-06-04

**Authors:** 


### THE FEDERATION OF MEDICAL AND ALLIED SOCIETIES.

At the annual dinner of this Federation, which was held on May 26 at the Café Royal, there was a distinguished and remarkably comprehensive gathering, in all some thirty-eight different societies being represented. The chief guests of the evening, Dr. Addison and his successor, Sir Alfred Mond, both made highly interesting speeches, and expressed good wishes for the success of a Federation the principles of which undoubtedly provide for expression of considered scientific and medical opinion on matters of great national importance. Dr. Addison particularly expressed his appreciation of the encouragement he had received from people who understood his work, and his audience on this occasion showed full appreciation of the exceptional difficulties which recently faced him. Fate decreed that many excellent schemes, conceived in a time of great national sympathy, in reconstructive measures should make their initial appearance at a time of extreme financial depression. They also met a phase of public opinion, not remarkable for any high degree of foresight, concerning the benefit, hygienic and financial, to be ultimately derived from the relatively small sums which it was proposed to invest in preventive medicine. It must have been eminently satisfactory to the late Minister of Health to realise, at this dinner, that the antagonism to his ideals, but lately expressed in many quarters, is not shared by the members of his own profession. Such modifications of policy as the speech of his successor may have foreshadowed do not, apparently, affect the purely medical aspect. The work which Dr. Addison originally suggested must be carried out. Its ultimate accomplishment may be regrettably delayed for financial reasons, but we are certain that his successor realises the essential soundness of his preventive policy.

In any case, a Health Minister must have available the opinion of the medical and allied professions. The views of all those professions represented on May 26 will, we have every confidence, soon be heard as the true "collective voice." The formation of this Federation has been urgently needed, and many of the most eminent professional men in the land are now associated with its organisation. Relatively few professional bodies still stand aside from the round-table discussion which it provides. May we urge that their hesitation should continue no longer, since no association becoming affiliated to the Federation in any sense loses its autonomy? It took many years to achieve that collectiveness resulting in the Royal Society of Medicine. Who can say to-day that that co-ordination of scientific bodies and scientific opinions has been a failure?

